# Pollutant Exposures from Natural Gas Cooking Burners: A Simulation-Based Assessment for Southern California

**DOI:** 10.1289/ehp.1306673

**Published:** 2013-11-05

**Authors:** Jennifer M. Logue, Neil E. Klepeis, Agnes B. Lobscheid, Brett C. Singer

**Affiliations:** 1 Indoor Environment Group, and; 2 Residential Building Systems Group, Environmental Energy Technologies Division, Lawrence Berkeley National Laboratory, Berkeley, California, USA; 3 Department of Civil and Environmental Engineering, Stanford University, Stanford, California, USA; 4 Center for Behavioral Epidemiology and Community Health (C-BEACH), Graduate School of Public Health, San Diego State University Research Foundation, San Diego State University, San Diego, California, USA

## Abstract

Background: Residential natural gas cooking burners (NGCBs) can emit substantial quantities of pollutants, and they are typically used without venting range hoods.

Objective: We quantified pollutant concentrations and occupant exposures resulting from NGCB use in California homes.

Methods: A mass-balance model was applied to estimate time-dependent pollutant concentrations throughout homes in Southern California and the exposure concentrations experienced by individual occupants. We estimated nitrogen dioxide (NO_2_), carbon monoxide (CO), and formaldehyde (HCHO) concentrations for 1 week each in summer and winter for a representative sample of Southern California homes. The model simulated pollutant emissions from NGCBs as well as NO_2_ and CO entry from outdoors, dilution throughout the home, and removal by ventilation and deposition. Residence characteristics and outdoor concentrations of NO_2_ and CO were obtained from available databases. We inferred ventilation rates, occupancy patterns, and burner use from household characteristics. We also explored proximity to the burner(s) and the benefits of using venting range hoods. Replicate model executions using independently generated sets of stochastic variable values yielded estimated pollutant concentration distributions with geometric means varying by < 10%.

Results: The simulation model estimated that—in homes using NGCBs without coincident use of venting range hoods—62%, 9%, and 53% of occupants are routinely exposed to NO_2_, CO, and HCHO levels that exceed acute health-based standards and guidelines. NGCB use increased the sample median of the highest simulated 1-hr indoor concentrations by 100, 3,000, and 20 ppb for NO_2_, CO, and HCHO, respectively.

Conclusions: Reducing pollutant exposures from NGCBs should be a public health priority. Simulation results suggest that regular use of even moderately effective venting range hoods would dramatically reduce the percentage of homes in which concentrations exceed health-based standards.

Citation: Logue JM, Klepeis NE, Lobscheid AB, Singer BC. 2014. Pollutant exposures from natural gas cooking burners: a simulation-based assessment for Southern California. Environ Health Perspect 122:43–50; http://dx.doi.org/10.1289/ehp.1306673

## Introduction

Natural gas cooking appliances are present in about half of the roughly 12 million housing units in California [California Energy Commission (CEC) 2004]. Nationally, 34% of households report using natural gas as their primary cooking fuel (U.S. Energy Information Administration 2005). Gas cooking burners emit air pollutants that can affect residential indoor air quality and increase health risks. Emitted pollutants include nitrogen dioxide (NO_2_), carbon monoxide (CO), and formaldehyde (HCHO).

At elevated ambient concentrations, NO_2_ has been associated with exacerbation of asthma (Hajat et al. 1999) and an increase in daily deaths (Touloumi et al. 1997). At higher concentrations, NO_2_ has been associated with increased sensitivity to allergens in patients with asthma (Tunnicliffe et al. 1994). Increased indoor NO_2_ concentrations from gas cooking have been associated with adverse health effects such as wheezing and decreased respiratory function (Jarvis et al. 1998).

Many studies have examined gas appliance-related concentrations of NO_2_ (Spengler et al. 1994; Yang et al. 2004) and CO (Akland et al. 1985; Fortmann et al. 2001) in homes. Measurement-based studies are imperative for understanding the physical properties that govern concentrations and exposures in homes; however, the costs and logistics of large-scale monitoring are barriers to using this method to quantify population-wide impacts.

The goal of this study was to estimate the impact of cooking with natural gas burners on in-home exposures to NO_2_, CO, and HCHO across a representative sample of homes in Southern California. Particulate matter (PM) mass emissions, especially ultrafine particles (diameter < 100 nm), are also a source of health concerns from natural gas burners; however, PM was not addressed in the present study.

To accomplish this analysis, we developed and utilized a population impact assessment modeling (PIAM) approach. The PIAM approach applies physics-based simulation model(s) to estimate one or more environmental or energy performance parameters for each home in a sample cohort selected or developed to represent a population. A key feature of the approach is that sample cohorts are developed from representative databases such as the Residential Energy Conservation Survey or the American Housing Survey (AHS) at the U.S. national level or the Residential Appliance Saturation Survey (RASS) in California. Home and occupant characteristics needed for the model but not available in these data sets were assigned based on independently determined relationships between these unspecified characteristics and data that are included in the databases or from other available data sources. Estimates for the individual homes were compiled to estimate population impacts. The approach can be applied at varying temporal or spatial scales; in a recent application, we examined the impact of air sealing and ventilation on annual energy use for homes across the United States (Logue et al. 2013).

The PIAM approach was applied to estimate in-home pollutant concentrations and exposures for Southern California households that have and use natural gas cooking burners. A mass-balance model was used to estimate time-dependent pollutant concentrations within each home for typical weeks in summer and winter. Age-based occupancy patterns and factors accounting for proximity to the cooking burners were used to estimate exposure concentrations over selected time durations for each occupant of each home. Time durations were selected to align with acute health-based standards (1 hr and 8 hr) and over each simulated 1-week period as an indicator of chronic exposures. Results across all simulated homes were aggregated to estimate distributions for the population. The potential impact of routine use of venting range hoods also was assessed.

## Methods

*Indoor air model*. The core component of the PIAM approach used for this analysis was a single-zone mass-balance model that simulates the emissions, dilution, deposition to surfaces, and removal by air exchange of air pollutants produced by residential cooking burners. The indoor air model uses the following governing mass-balance equation:

*V*(*dC*_in,i_/*dt*) = *E*_i_ – *k*_i_*VC*_in,i_ – *aVC*_in,i_ + *aVP*_i_*C*_out,i_ *.* [1]

In this equation, written for pollutant species i, *V* is residence volume (in cubic meters), *C*_in,i_ is the indoor concentration (in micrograms per cubic meter), *E*_i_ is the emission rate (in micrograms per hour), *k*_i_ is the first-order deposition rate constant (per hour), *a* is the air exchange rate (AER) (per hour), *P*_i_ is penetration efficiency—the fraction of pollutant retained as air enters from outdoors, *t* is time (hours), and *C*_out,i_ is the outdoor concentration (in micrograms per cubic meter). *E*_i_ was selected from emission factors (in nanograms per Joule) measured in a recent study of U.S. cooking ranges by Singer et al. (2010). Fuel use for cooktop burners was set at 1.23 × 10^5^ J/min (7 kBtu/hr) as an estimated time-averaged mean. An oven-specific fuel use algorithm was developed based on measurements of actual oven firing patterns as described below. *P* was assumed to be 1 for all pollutants modeled in the present study.

Deposition was assumed to be negligible for CO and HCHO but not NO_2_. Although HCHO is known to reversibly sorb to indoor materials, the overall rate coefficients for adsorption and desorption in furnished homes (Sherman and Hult 2013; Xu and Zhang 2003) appear to be much slower than air exchange for all but the lowest ventilation rates considered in the present study. The NO_2_ first-order deposition rate varies with humidity and surface characteristics, and reported values of the first-order rate constant for furnished homes vary from 0.11 to 1.4/hr (Nazaroff et al. 1993; Spengler et al. 1994; Spicer et al. 1993; Yang et al. 2004). Yang et al. (2004) estimated a representative deposition rate of 1.05/hr for western countries. This estimate is on the higher end of the range of values in the literature. Simulations were run with *k*_i_ values of 1.05/hr and 0.5/hr to encompass what we assessed to be the upper and lower bounds on the median value across California homes.

Equation 1 was adapted into Equations 2 and 3 to separately track indoor pollutants originating from indoor emissions (*C*_in_I,i_) and indoor pollutants from outdoor sources (*C*_in_O,i_,) with total concentrations calculated as the summed contributions from the two sources (Equation 4).

*d*(*C*_in_I,i_)/*dt* + (*k*_i_ + *a*)*C*_in_I,i_ – *E*_i_ /*V* = 0, [2]

*d*(*C*_in_O,i_)/*dt* + (*k*_i_ + *a*)*C*_in_O,i_ – *aC*_out,i_ = 0, [3]

and

*C*_in,i_ = *C*_in_I,i_(*t*) + *C*_in_O,i_(*t*). [4]

Equations 2 and 3 can each be solved recursively for *C*_in_I,i_ and *C*_in_O,i_, respectively, with any of the parameters held constant within a given time step and allowed to vary from one time step to another. Equation 5 presents the recursive solution for the indoor concentration resulting from gas burner emissions:

*C*_in_I,i_(*t*) = [*C*_in_I,i_(*t –* 1)exp^–(^*^a^*^(^*^t^*^) +^ *^k_i_^*^)Δ^*^t^*] *+* {[*E*_i_(*t*)(1 – exp^–(^*^a^*^(^*^t^*^) +^ *^k_i_^*^)Δ^*^t^*)] ÷ [(*a*(*t*) + *k*_i_)*V*]}. [5]

In this equation, *C*_in_I,i_ is the indoor concentration of pollutant *i* generated from appliance use at time *t* and at the previous time step (*t –* 1), Δ*t* is the time interval (set at 1 min), and *E* and *V* are the emission rate and residence volume, as defined above. Equation 6 is the solution for the indoor concentration of pollutant *i* originating from outdoors:

*C*_in_O,i_(*t*) = [*C*_in_O,i_(*t –* 1)exp^–(^*^a^*^(^*^t^*^) +^ *^k_i_^*^)Δ^*^t^*] *+ C*_out,i_(*t*){[*a*(*t*)(1 – exp^–(^*^a^*^(^*^t^*^) +^ *^k_i_^*^)Δ^*^t^*)] ÷ [*a*(*t*) + *k*_i_]}. [6]

**Figure 1:**
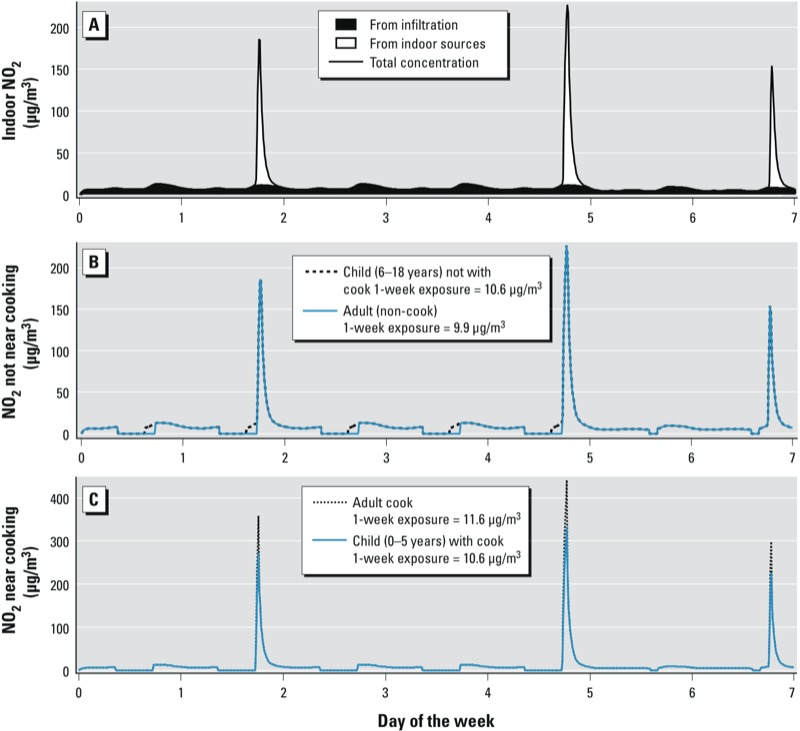
Example results: simulated time-resolved NO_2_ concentrations in a 36-year-old, 1,125‑ft^2^ home with four occupants (one 0–5, one 6–18, and two 35–54 years of age) for 1 week in winter. (*A*) Indoor concentration of NO_2_ originating from indoor and outdoor sources. (*B*) Simulated exposure concentration experienced by the two occupants assumed to not be near the cooking activity (*F*_prox_ = 1). (*C*) Simulated exposure concentration for the cook (*F*_prox_ = 2) and a small child assumed to be near the cooking (*F*_prox_ = 1.5).

The recursive model was implemented and solved with code written in the R programming environment (R Development Core Team 2011). The primary outputs were indoor concentrations of NO_2_, CO, and HCHO for typical summer and winter weeks, at 1-min resolution. We estimated time-averaged indoor and exposure concentrations (concentrations experienced by individual occupants) over durations corresponding to acute health-based standards (1 hr and 8 hr) and over each 1-week simulation. Exposure concentrations were estimated for each occupant considering only their pollutant intake in the home. For example, if in a given simulation an occupant remained at home for just 30 min after cooking was started and the time-averaged concentration of CO over those 30 min was 20 ppm, the 1-hr exposure concentration was calculated as 10 ppm. Figure 1 provides example plots of NO_2_ in-home concentrations and exposure concentrations for three occupants of one home during a simulated week in winter.

*Model implementation*. We applied the PIAM approach to study a cohort of Southern California homes that included representative variations in the characteristics that impact pollutant emissions from cooking burners and the associated occupant exposures. We simulated 2 weeks of activity—one in summer and one in winter—for each residence in our sample. Only emissions from natural gas combustion, not emissions from cooking food, were included. The Southern California Region (SoCal) comprises the following six counties: Los Angeles, San Bernardino, Riverside, San Diego, Orange, and Ventura. We obtained distributional information about homes in this area from the publicly available 2003 RASS database, which contains anonymous data for almost 22,000 households throughout California (CEC 2004). The SoCal cohort taken from the 2003 RASS consists of 6,634 households containing 19,464 individual residents. The RASS data set reflects the variability in home sizes and types seen in California. The RASS provides a weighting for each home in the database to construct a statistically representative sample of the population served by the four largest California utilities. Applying these weightings, the modeled SoCal cohort represents 3,560,000 homes and 11,680,000 occupants. The population estimates presented here reflect these weighting values. The RASS contains information about building type, age, volume, location, household demographics, cooktop type, and the frequency of cooking with the cooktop or oven. We used the 2003 RASS data set (collected in 2002 through 2003) because it includes meal-specific cooking frequencies that were not collected in the 2009 RASS (CEC 2004). Our study sample included residences that reported using a gas cooktop or oven (excluding homes that used bottled gas to cook) at least once during the course of a typical week. In the Southern California Region, 56% of homes reported using natural gas (excluding the 2% reported using bottled gas).

Required activity factors were assigned to each sample home based on household-specific data available in the RASS and data from other published surveys and reports. Cooking frequency was taken directly from the RASS. Meal-specific cooking durations and burner selections were calculated from a web-based cooking survey (Klug et al. 2011b). The survey included responses from 372 participants predominately in California and included questions relating to home, household, and cooking appliance characteristics and weekly patterns of meals cooked. This survey provided meal-specific data on the frequency of oven use, number of cooktop burners used, and duration of burner use. Based on the cooking survey, the model assumes one cooktop burner for breakfast or lunch and two cooktop burners for dinner and also includes oven use for all dinners cooked in half of the homes. The duration of each discrete cooking event was assigned by sampling from lognormal distributions of cooktop and oven use duration for the specific meal (breakfast, lunch, dinner), based on data collected in the cooking survey (Klug et al. 2011b); the distribution summary statistics are provided in Supplemental Material, Table S1. We used the median reported data from the National Human Activity Patterns Survey (NHAPS) (Klepeis et al. 2001) to establish meal times and to establish archetypal home occupancy patterns based on occupant age (0–5, 6–17, 18–64, ≥ 65 years) and weekend or weekday (for specific assignments, see Supplemental Material, Table S2). Cooking burner emission factors for NO_2_, CO, and HCHO were based on measurements reported by Singer et al. (2010) for twelve ranges, each including a cooktop and oven. Each home was randomly assigned the emission factors from one cooktop and one oven from the data set and those emission factors were used for all modeling of the home.

AER. Distributions of empirical AERs were developed from studies reporting AER measurements in Southern California homes (Health Effects Institute 2010; Offerman 2009; Wilson et al. 1993, 2003). Distributions were developed for winter and non-winter seasons for three home age ranges by date of construction (pre-1980, 1981–1995, and post-1995). We randomly sampled from these distributions to select a winter AER and a summer AER for each home based on home age. Summer AERs were higher, likely due to more window opening. Higher summer AERs result in lower modeled concentration estimates in summer compared with winter. Relative to the 2003 RASS database, the current (ca. 2013) California housing stock includes newer homes with lower AERs. Lower AERs translate to less dilution and higher concentrations of pollutants from indoor sources.

Outdoor air pollutants. Typical outdoor NO_2_ and CO profiles were developed for each county for a winter week and a summer week based on concentrations measured at ambient air quality monitoring sites. Data were downloaded from the U.S. Environmental Protection Agency (EPA) AirData website (U.S. EPA 2012a). A representative monitoring site was selected for each county and all homes in that county were assumed to have the same outdoor concentrations. If more than one monitoring site existed in a county, we selected the site that reported the median annual average concentration from among the available sites reporting data from the county. Hourly outdoor profiles for each site were developed by calculating the average concentrations from all available data from 2008–2009 by hour and by day of the week. Whereas indoor concentrations of NO_2_ and CO can be dominated by contributions from outdoor air and unvented indoor combustion sources, indoor HCHO concentrations typically depend on a wider variety of sources, including material emissions, chemical reactions, outdoor sources, and indoor combustion (Salthammer et al. 2010; Zhang et al. 1994). We did not incorporate HCHO from other indoor or outdoor sources into the analysis; therefore, our estimates of indoor HCHO concentrations reflect only the incremental contribution of NGCB exhaust.

Estimated pollutant concentrations were linked to archetypal patterns of home occupancy according to age group (0–5, 6–18, 19–64, ≥ 65 years) for each individual residing in each modeled home (Klepeis et al. 2001); this was done to explore the impact of age-based activity patterns on individual-level exposures. When occupants were not home based on occupancy profiles, their exposure concentrations were assumed to be zero.

Proximity factors. The model accounts for elevated concentrations of NGCB pollutants in the kitchen relative to other parts of the home (Berwick et al. 1989; Hoek et al. 1984; Noy and Lebret 1986; Palmes et al. 1977, 1979) and assumes that anyone in the kitchen during cooking will be exposed to these higher concentrations. We account for this proximity effect by assigning one adult cook for each cooking event and by assuming that any young children (0–5 years of age) present in the home during cooking are nearby. Exposures are calculated by multiplying the estimated indoor-generated pollutant concentration by proximity factors (*F*_prox_) of 2.0 for the cook and 1.5 for children 0–5 years of age, then adding the contribution from outdoor sources, which was assumed to be uniform throughout the home. Proximity factors were determined by reviewing published data on burner-related pollutant concentration in kitchens and other areas of the home and determining ratios of concentrations measured in kitchens compared with other rooms (Berwick et al. 1989; Garrett et al. 1999; Hoek et al. 1984; Zota et al. 2005) and near source compared with away from source (McBride et al. 1999).

Sensitivity and uncertainty analysis. As described above, the model assigns values for key characteristics that are not specified in the RASS. Parameters that are assigned as non-varying for the week are the AER, cooktop and oven pollutant emission rates, whether the oven was used, specific days of week during which meals were prepared, number of cooktop burners used for each meal, start time of each meal on weekend days and weekdays, and which adult was the cook. The RASS database includes the self-reported frequency of breakfast, lunch, and dinner cooked during each week for each home. We randomly assigned days for those meals to be cooked during the week. The burner duration was assigned anew for each meal as described previously.

To evaluate the sensitivity of model results to these assigned values, we executed the model 15 times to simulate a winter week for the entire sample cohort, using the higher NO_2_ first-order deposition rate constant (*k*_i_ = 1.05/​hr) and with all parameters assigned anew for each model execution. We evaluated the consistency of results across model executions by calculating the variation in the geometric mean (GM), geometric standard deviation (GSD), and summary statistics (5th, 25th, 50th, 75th, and 95th percentiles) of the calculated distributions of in-home and exposure concentrations.

To assess the impact of proximity factors, we also estimated exposures assuming that all occupants are exposed only to the time-dependent concentrations calculated for the home (*F*_prox_ = 1 for all occupants) for the winter week, using the higher NO_2_ first-order deposition rate constant (*k*_i_ = 1.05/hr). This assumes that being in the same room or adjacent to cooking has no impact on exposure.

Range hood pollutant mitigation analysis. We conducted an additional analysis to assess the potential benefits of widespread and routine use of vented range hoods. We showed previously that use of a vented range hood can dramatically reduce concentrations of pollutants from cooking burners (Delp and Singer 2012). Although the majority of California homes appear to have an installed range hood, it is unknown what fraction of these are vented as opposed to recirculating (Klug et al. 2011a), and available data suggest that a minority of households routinely use range hoods during all cooking (Klug et al. 2011b; Mullen et al. 2012). We simulated vented hood use by reducing all pollutant emission rates by 55%; this value reflects the mean capture efficiency reported in a measurement-based study of range hoods installed in California homes (Singer et al. 2012).

*Pollutant standards*. We used ambient air quality standards set by the U.S. EPA (2012b) and the California EPA and guidelines established by California’s Office of Environmental Health Hazard Assessment (OEHHA) as guides to set benchmarks for undesirable levels of indoor air pollutants (Table 1). Acute standards have averaging times of 1 hr for NO_2_, CO, and HCHO, and 8 hr for CO and HCHO. Chronic (annual average) concentration limits are available for NO_2_ and HCHO. We defined an exceedance as occurring when the indoor household concentration, or an individual occupant’s exposure, exceeded one of the benchmark levels noted in Table 1. We note, however, that outdoor standards can be strictly exceeded only when outdoor concentrations exceed those standards.

**Table 1:** Pollutant standard and guideline concentrations for various exposure periods.

Pollutant	1-hr average (acute)	8-hr average (acute)	Annual average (chronic)	Standard (reference)
NO_2_	180 ppb (339 μg/m^3^)	NA	30 ppb (57 μg/m^3^)	CAAQS (CARB 2010)
	100 ppb (188 μg/m^3^)	NA	53 ppb (100 μg/m^3^)	NAAQS (U.S. EPA 2012b)
CO	20 ppm (23 mg/m^3^)	9 ppm (10 mg/m^3^)	NA	CAAQS (CARB 2010)
	35 ppm (40 mg/m^3^)	9 ppm (10 mg/m^3^)	NA	NAAQS (U.S. EPA 2012b)
HCHO	45 ppb (55 μg/m^3^)	7.3 ppb (9 μg/m^3^)	7.3 ppb (9 μg/m^3^)	Non-cancer REL (OEHHA 2007)
Abbreviations: CAAQS, California Ambient Air Quality Standard; CARB, California Air Resources Board; NA, not available; NAAQS, National Ambient Air Quality Standard; OEHHA, California’s Office of Environmental Health Hazard Assessment; REL, reference exposure level.				

*Scenarios*. The simulation model was executed to estimate distributions of in-home concentrations and exposures for the population of Southern California homes using line-distributed natural gas cooking burners at least once per week. Results are presented for simulated cooking over 1 week according to five scenarios:

Scenario 1: winter, proximity effect included (*F*_prox_ = 2.0 for cook, 1.5 for 0- to 5-year-olds), no range hood use
Scenario 2: summer, proximity effect included, no range hood use
Scenario 3: winter, no proximity effect (*F*_prox_ = 1.0 for all), no range hood use
Scenario 4: winter, proximity effect included, all homes use range hoods with 55% capture efficiency
Scenario 5: summer, proximity effect included, all homes use range hoods with 55% capture efficiency.

Summary statistics for scenario 1 with *k*_i_ = 1.05/hr for NO_2_ are presented as a mean ± range to indicate the variation across the 15 replicate runs that resulted from reassigning parameter values. The range is the difference between the mean value and the run with the largest difference, higher or lower, from the mean value. Hereafter, all uses of the “±” notation present the range of values for the 15 replicate runs for scenario 1.

## Results

**Figure 2:**
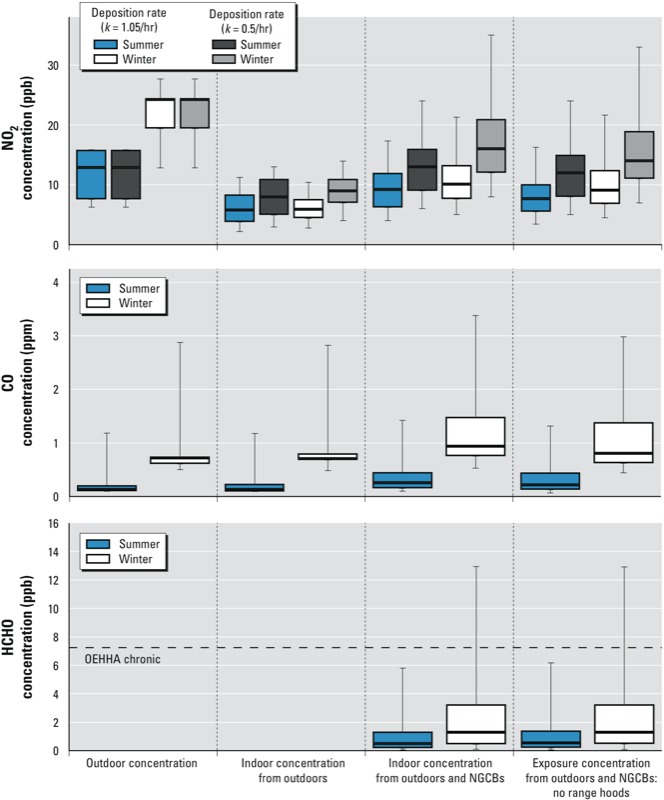
One-week time-averaged indoor pollutant concentrations estimated by simulation modeling for weighted sample of 6,634 of SoCal homes. Both summer (scenario 2) and winter (scenario 1) results presented in this figure assume no range hood use and apply near-source proximity factors to estimate exposure concentrations. Boxes indicate 25th (bottom), 50th (line within box), and 75th (top) percentiles; whiskers represent 5th and 95th percentiles. Dashed horizontal lines are standards from Table 1 that are within ranges shown on graphs. Results presented for scenario 1 are the mean values from 15 model executions. See Supplemental Material, Table S3, for tabulated results.

Figure 2 shows the estimated distributions of 1-week average household pollutant concentrations and exposure concentrations to HCHO from gas cooking burners. Figure 2 also shows distributions for NO_2_ and CO concentrations and exposure concentrations from gas burners plus infiltration of outdoor pollutants. Estimated distributions are shown for typical winter and summer weeks for scenarios in which venting range hoods were not used (i.e., for scenarios 1 and 2). The data used to construct Figure 2 are provided in Supplemental Material, Table S3. Estimated exposure concentrations differ from the household concentrations because they account for proximity factors for cooks and small children and also account for some people not being home during all periods when indoor concentrations are elevated. Because most occupants were assumed to be out of the home for 9 hr on weekdays and 2 hr on weekends, occupancy patterns reduced the weekly exposure concentrations relative to the household concentrations.

Indoor concentrations of NO_2_ from either indoor or outdoor sources depend strongly on the rate constant of indoor deposition, the specified *k*_i_ values for NO_2_. A higher *k*_i_ value assumes faster removal by deposition, resulting in lower indoor concentrations. The median estimated weekly average indoor NO_2_ concentration for the sample of home simulations was lower than the outdoor concentration, predominately due to deposition losses indoors.

The simulation model calculated the contribution of NGCBs to the total weekly average concentration estimated for each modeled home. Across the various scenarios, the simulation model estimates that NGCBs had average contributions of 25% (summer, *k*_i_ = 1.05/hr), 33% (summer, *k*_i_ = 0.5/hr), 35 ± 1% (winter, *k*_i_ = 1.05/hr), and 39% (winter, *k*_i_ = 0.5/hr) to the week-averaged indoor NO_2_ concentrations. Cooking burners contributed on average 30% and 21 ± 1% of the estimated indoor week-averaged concentrations of CO in the cohort in summer and winter respectively. Cooking burner contributions to indoor concentrations were smaller in the summer because of higher AERs. The simulation model estimated that cooking burners would not yield indoor concentrations above chronic standards for NO_2_ or CO for the scenarios evaluated (Figure 2). Modeled homes had an estimated median increase in week-average HCHO concentrations due to NGCBs on the order of 1 ppb. This is an order of magnitude lower than concentrations measured over multiple days in homes. Logue et al. (2011) aggregated multiple studies that reported measured HCHO concentrations in homes and reported a median measured concentration of 19 ppb. This is consistent with expectations that the contribution of emissions from burners to chronic HCHO in most homes is small compared with emissions from building materials and furnishings. Nonetheless, for the scenarios evaluated, the model estimates that HCHO emissions from NGCBs alone, in the absence of other HCHO sources, would lead to exposures exceeding at least one chronic standard for 3–10% of occupants and in 3–9% of homes, depending on season (Figure 2). The lower bounds of these ranges are for summer weeks; the upper bounds are for winter weeks. Distributions are presented in Figure 2 for scenario 1 (winter) and scenario 2 (summer).

**Figure 3:**
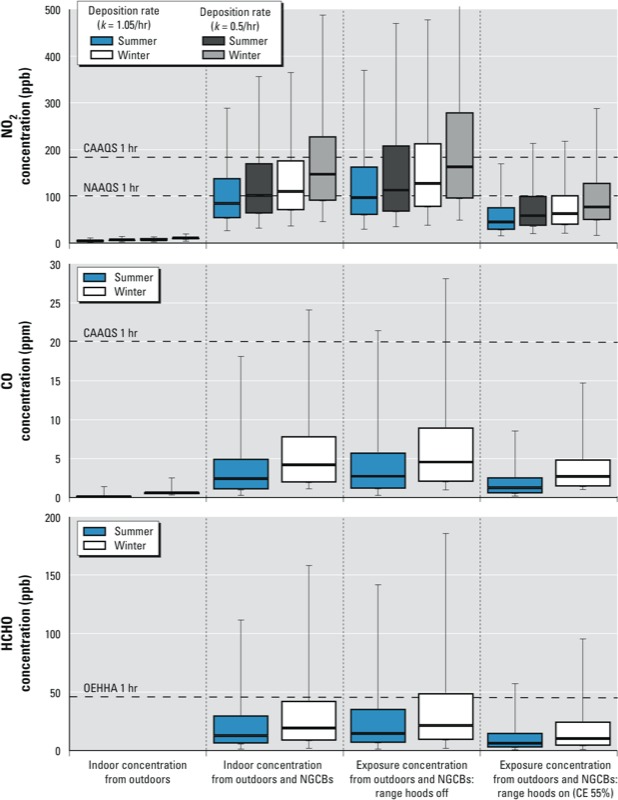
Highest 1-hr time-averaged indoor pollutant concentrations estimated by simulation modeling of the weighted sample of 6,634 SoCal homes and exposure concentrations for the weighted sample of 19,464 individual occupants. Estimated indoor concentrations presented for scenario 1 (winter) and scenario 2 (summer), both of which assume no range hood use. Estimated exposure concentrations presented in this figure all apply near-source proximity factors, with one pair of scenarios assuming no range hood use and the second pair of scenarios assuming use of a range hood with 55% capture efficiency (CE) during every cooking event. Boxes indicate 25th (bottom), 50th (line within box), and 75th (top) percentiles; whiskers represent 5th and 95th percentiles. Dashed horizontal lines are standards from Table 1 that are within ranges shown on graphs. Results presented for scenario 1 are the mean values from 15 model executions. See Supplemental Material, Table S3, for tabulated results.

Simulation model results suggest that acute air pollutant concentration standards are commonly exceeded in homes that use NGCBs without venting range hoods. Figure 3 shows distributions of the estimated maximum hourly concentration for the homes simulated as well as estimated maximum hourly exposure concentrations for the occupants simulated. The estimated contributions of outdoor pollutants to peak 1-hr indoor concentrations were negligible compared with model estimates of maximum 1-hr concentrations, indicating the importance of indoor sources to acute exposures (Figure 3). The simulation model estimates that in homes that cook with unvented NGCBs, a large proportion of residences are exposed to concentrations that exceed the 1-hr standard for NO_2_. The estimated fraction of residents exposed to a concentration exceeding the 1-hr standard varies by season and with the assumed NO_2_ deposition rate constant (*k*_i_ value), with estimates ranging from 41% to 70% (Table 2). The model produced a similar range of estimates (27–54%) for the number of occupants exposed to HCHO levels exceeding an acute standard and a smaller but sizeable fraction (4–9%) exposed to CO concentrations that exceed acute CO standards. Model estimates suggest that the majority of exceedences of the 1-hr standard are due to indoor emissions from NGCBs (Table 2). The model estimated that the mean number of acute exceedances per week among homes in which exceedances occurred ranged from 2.4 for summer to 3.6 for winter scenarios without range hood use, depending on the pollutant and season (Table 2).

**Table 2:** Model-estimated frequencies of pollutant concentrations exceeding acute health-based pollutant standards in homes that use natural gas cooking burners at least once per week (*n* = 6,634).

Pollutant in homes	Summer		Winter	
	No hood	With hood	No hood	With hood
NO_2_ (*k* = 1.05/hr)
Exceedances of 1-hr NAAQS standard
Homes with exceedance (%)	41	10	55 ± 2^*a*^	18
Homes with exceedance due to indoor emissions only (%)	38	9	51 ± 1	15
Mean exceedances per home exceeding (*n*/week)^*b*^	3.0	2.5	3.4 ± 0.1	2.4
NO_2_ (*k* = 0.5/hr)
Exceedances of 1-hr NAAQS standard
Homes with exceedance (%)	51	17	70	30
Homes with exceedance due to indoor emissions only (%)	47	15	64	24
Mean exceedances per home exceeding (*n*/week)	3.3	2.6	3.6	2.7
CO
Exceedances 1-hr CAAQS standard
Homes with exceedance (%)	4	0.4	7 ± 1	2
Homes with exceedance due to indoor emissions only (%)	4	0.4	6 ± 1	1
Mean exceedances per home exceeding (*n*/week)	2.4	1.2	2.6 ± 0.5	2.4
Exceedances of 8-hr NAAQS standard
Homes with exceedance (%)	2	0.2	8 ± 1	2
Mean exceedances per home exceeding (*n*/week)	2.6	2.1	2.5 ± 0.3	1.9
HCHO
Exceedances of 1-hr OEHHA guideline
Homes with exceedance (%)	15	4	24 ± 2	11
Mean exceedances per home exceeding (*n*/week)	3.2	2.6	3.1 ± 0.3	2.6
Exceedances of 8-hr OEHHA guideline
Homes with exceedance (%)	27	12	52 ± 2	30
Mean exceedances per home exceeding (*n*/week)	3.3	2.6	3.5 ± 0.1	3.2
The two sets of NO_2_ data reflect the two first-order loss rates (*k*_*i*_) used to simulate NO_2_ dynamics. ^***a***^Summary statistics for scenario 1 (only for *k*_*i*_ = 1.05/hr for NO_2_) are presented as a mean ± range to indicate the variation across the 15 replicate runs that resulted from reassigning parameter values. The range is the difference between the mean value and the run with the largest difference, higher or lower, than the mean value. ^***b***^"Mean exceedences per home exceeding" indicates the mean number of times a home that exceeded the specified standard at least once exceeded that standard during the simulated week.				

Table 3 presents estimated personal exposure exceedances for acute health-based standards by age group and for the assigned cooks for the simulated week during the winter. Table 3 includes estimated distributions from a simulation model execution that did not account for differences in exposure according to proximity to the kitchen (scenario 3) and for a model execution that accounted for differences in proximity but assumed the home had a range hood operating during all cooking events (scenario 4). Independent of the age group and cook status, if the model indicated that someone experienced an exceedance, on average they experienced 2–3 exceedances over the modeled week. When the proximity factors were applied, the sub-groups with the greatest likelihood to experience acute exceedances were cooks followed by 0- to 5-year-olds. Cooks also had the largest difference between the percentage of individuals estimated to experience an exceedence when proximity factors were applied (Table 3, winter, differences by proximity) and when proximity was assumed to have no effect (Table 3, winter, no differences by proximity). The age group of 6- to 18-year-olds had the lowest percentage of occupants exceeding the standard because they were assumed to not cook or be in the kitchen at the time of cooking events. Variations in modeled concentrations between runs with and without proximity factors for non-cooks and those who are not 0–5 years of age are due to differences in initial parameters selected for each model run.

**Table 3:** Model-based estimates of the percentage of occupants that would be exposed to a time-averaged concentration exceeding an acute health-based pollutant standard during a typical winter week.

Age group^**^	Percentage of SoCal population (%)	NO_2_ (*k* = 1.05/hr)	NO_2_ (*k* = 0.5/hr)	CO		HCHO	
		1-hr (%)	1-hr (%)	1-hr (%)	8-hr (%)	1-hr (%)	8-hr (%)
No hood, differences by proximity
0–5	11.4	72 ± 6	80	11 ± 4	11 ± 3	29 ± 6	57 ± 6
6–18	21.8	53 ± 4	63	6 ± 3	7 ± 2	21 ± 4	50 ± 5
19–64	58.5	63 ± 2	74	9 ± 1	9 ± 2	26 ± 4	53 ± 3
≥ 65	8.23	65 ± 3	76	9 ± 2	8 ± 2	26 ± 7	53 ± 3
Cook	30.5	76 ± 2	83	13 ± 1	11 ± 2	33 ± 4	57 ± 2
No hood, no differences by proximity
0–5	11.4	58	74	8	9	27	56
6–18	21.8	54	66	7	8	25	52
19–64	58.5	54	69	8	9	25	53
≥ 65	8.23	47	66	4	7	21	48
Cook	30.5	54	70	8	8	25	53
With hood, differences by proximity
0–5	11.4	34	50	3	3	17	34
6–18	21.8	15	25	1	3	14	31
19–64	58.5	29	38	3	3	15	32
≥ 65	8.23	27	40	1	1	11	29
Cook	30.5	43	52	4	3	18	35
Age group includes cook as a separate class. First two groups of results assume no range hood use; middle group assumes no proximity effect. Summary statistics for scenario 1 (only for *k*_*i*_ = 1.05/hr for NO_2_) are presented as a mean ± range to indicate the variation across the 15 replicate runs that resulted from reassigning parameter values. The range is the difference between the mean value and the run with the largest difference, higher or lower, than the mean value.							

We performed 15 separate simulations for scenario 1 to evaluate the influence of parameter selection on model-estimated distributions of concentrations in homes. The 15 separate simulations produced consistent results as indicated by the statistics of calculated in-home and exposure concentration distributions; results are presented in Supplemental Material, Table S3. GMs, 25th, 50th, and 75th percentile values varied by < 10%. Statistics at the tails (5th and 95th percentiles) varied by < 20%. The repeated simulations produced ranges of estimates that were relatively narrow in relation to the median values across the simulations. Results are shown in Tables 2–3 for the winter week when no hood was used (using *k*_i_ = 1.05/hr for NO_2_ deposition and accounting for differences in proximity). Although we did not evaluate the magnitude of uncertainty for other scenarios, we assumed that it would be comparable to that observed for scenario 1 (with *k* = 1.05/hr for NO_2_ deposition). The only sampled distribution that varied between runs was the sample AER for summer versus winter. While the GMs of these distributions differ, the GSDs are similar for each home age group and will result in similar uncertainties.

The results presented so far were for homes that did not use a venting range hood when cooking. We explored the benefit of all homes having and using venting range hoods for all cooking events by reducing mass emission rates by 55%, an estimate of mean effectiveness (Singer et al. 2012). The impact of universal use of moderately effective range hoods on the estimated maximum 1-hr in-home and exposure concentrations is shown in Figure 3 and Table 2. Table 2 shows that the estimated percentages of homes exceeding the most conservative 1-hr acute standard decreased from 55% to 18% for NO_2_ with *k*_i_ = 1.05/hr, from 70% to 30% for NO_2_ with *k*_i_ = 0.5/hr, from 7% to 2% for CO, and from 24% to 11% for HCHO, compared with homes that did not use range hoods in winter. In summer simulations, the estimated percentage of homes exceeding the most conservative 1-hr acute standard decreased from 21% to 10% for NO_2_ with *k*_i_ = 1.05/hr, from 51% to 17% for NO_2_ with *k*_i_ = 0.5/hr, from 4% to 0.4% for CO, and from 15% to 4% for HCHO, compared with homes that did not use range hoods. Table 3 presents analogous results showing substantial reductions in the estimated frequencies of individual occupant exposure concentrations exceeding standards.

## Discussion

There are many reports of residential NO_2_ measurements in California and other U.S. locations (Lee et al. 2002; Ryan et al. 1988; Spengler et al. 1983, 1994), yet few of these can be used to directly assess whether the simulation results of this study are consistent with current concentrations in homes. Many of the studies are decades old and outdoor concentrations were typically higher than those used in the present simulation study. The older studies sampled in homes with appliances that were different and may have had different emission factors than cooking appliances currently in use. Therefore, we compare our results to a recent measurement study of concentrations in California homes (Mullen et al. 2012) when possible and refer to other literature reports as necessary and warranted.

During November 2011 through March 2012, Mullen et al. (2012) measured pollutant levels over 6-day periods in 155 homes, mostly in Northern California. Their measured concentrations were on par with simulated concentrations in the present study. Among 117 homes that reported cooking with a gas appliance at least once during sampling, the time-integrated measurements had a fitted NO_2_ GM (GSD) of 12 ppb (2.2) in the bedroom and 15 ppb (2.3) in the kitchen. Time-integrated outdoor NO_2_ levels in the measurement study had a GM (GSD) of 14.1 ppb (1.8). The repeated winter simulations in the present study had a GM (GSD) of 10.1 ± 0.13 ppb (0.8 ± 0.02) when the higher first-order rate constant for deposition (*k*_i_ = 1.05/hr) was used and 16.2 ppb (1.6) for the single winter simulation with the lower NO_2_ deposition value (*k*_i_ = 0.5/hr). The outdoor NO_2_ for the simulated homes had a fitted GM (GSD) of 21.2 ppb (1.3). Valid time-resolved CO data were available for 116 of the homes in the measurement study. The GM (GSD) of the highest 1-hr CO was 3.1 ppm (4.2) for these data. Highest 1-hr CO levels in the simulation homes in the repeated winter simulations had a GM (GSD) of 4.2 ± 0.16 ppm (2.7 ± 0.11), and highest 1-hr CO due only to the gas burner emissions had a GM (GSD) of 2.6 ± 0.14 ppm (4.2 ± 0.23).

Wilson et al. (1995) measured CO concentrations in 277 California homes and reported median values for 1-hr and 48-hr averages of 3.0 and 1.2 ppm respectively. These values are comparable to our median estimates for highest 1-hr and 1-week average CO concentrations in Southern Califorina homes in winter of 4.2 ± 0.3 ppm and 0.9 ± 0.02 ppm, respectively. As shown in Figure 3 for the *k*_i_ = 1.05/hr scenarios, the model estimated a median value of highest 1-hr indoor NO_2_ concentrations across the sample cohort of 85 ppb for summer and 110 ± 3 ppb for winter. Estimated 5th and 95th percentile values were 27 ppb and 288 ppb for summer and 36 ± 2 ppb and 364 ± 31 ppb for winter. The only U.S. study reporting peak NO_2_ concentrations that we could find in the literature (Fortmann et al. 2001) reported peak NO_2_ during cooking that ranged from 40 to 150 ppb based on a single cooktop. Taken as a group, these comparisons suggest that the estimates from our modeling study are reasonable and generally consistent with available monitoring data.

The model did not include homogeneous or heterogeneous chemical reactions, such as the reaction of NO and ozone to produce NO_2_. These reactions would increase the effective NO_2_ emission rate of the cooktop, particularly in summer, which underscores that our estimates for NO_2_ concentrations in summer are conservative and that the health impact of NGCB is likely even larger than modeled here. The possible magnitude of this effect is discussed in the Supplemental Material, p. 2.

## Conclusions

Our results suggest that in homes using NGCBs without venting range hoods, a substantial proportion of occupants experience pollutant concentrations that exceed health-based standards and guidelines. Using simulations of Southern California households cooking at least once per week, we estimate that pollutant levels exceed ambient air quality standards for NO_2_ and CO in 55–70% and 7–8% of homes during a typical week in winter (Table 2). Approximately half of homes in California and 34% of homes nationally have natural gas cooking burners, and assuming that the critical parameters of pollutant emission rates from appliances, homes sizes, and cooking patterns have similar distributions throughout the state as occur in the SoCal cohort, we estimate through extrapolation that approximately 12 million and 1.7 million Californians routinely could be exposed to NO_2_ and CO levels, respectively, exceeding ambient air standards in a typical week in winter. Additional work is needed to estimate the frequencies at which air quality benchmarks are exceeded in the tens of millions of U.S. homes that have natural gas cooking burners.

The U.S. EPA and California outdoor health standards, NAAQS (U.S.EPA 2012b) and CAAQS (CARB 2010), respectively, are legally enforceable regulations. If outdoor concentrations exceed these standards in specific areas they are referred to as “nonattainment” areas. The health impacts of being in nonattainment are thought to be significant enough to warrant a wide array of fiscal and regulatory penalties to achieve compliance. Our model-based estimates suggest that during the winter in Southern California, 55–70% of homes that have and use natural gas burners without venting have indoor air pollution levels consistent with ambient outdoor levels in nonattainment areas.

The hazard posed by natural gas cooking burners can be mitigated substantially through the use of venting range hoods that capture cooking burner pollutants—as well as pollutants generated from cooking activities—at the point of emissions and exhaust them to the outdoors. The range hood modeled in the present study was assumed to have the average capture efficiency measured by Singer et al. (2012) in homes. Our estimates suggest that improving range hood effectiveness through changes in occupant behavior or by installing hoods that are quieter (and thus more likely to be used), or by improving their capture efficiency (Delp and Singer 2012), would greatly reduce the number of persons who may be exposed to indoor air pollutants at levels that exceed ambient air quality standards.

## Supplemental Material

(446 KB) PDF
