# Supplementary material for: Pollutant Exposures from Natural Gas Cooking Burners: A Simulation-Based Assessment for Southern California
Source: Environ Health Perspect. 2013 Nov 5;122(1):43–50. doi: 10.1289/ehp.1306673 (PMC3888569; doi:10.1289/ehp.1306673)
Supplement: (446 KB) PDF [file ehp.1306673.s001.508.pdf]

Environmental Health Perspectives doi:10.1289/ehp.1306673

Supplemental Material

Pollutant Exposures from Natural Gas Cooking Burners: A Simulation-Based Assessment for Southern California

Jennifer M. Logue, Neil E. Klepeis, Agnes B. Lobscheid, and Brett C. Singer

Table of Contents

Supplemental Material, Table S1. Properties of cooking duration distributions………………………..2

Supplemental Material, Table S2. Age-based time-location patterns based on NHAPS data (Klepeis et al. 2001)………………………………………………………………………………………….2

Indoor Reactions of NO and Ozone to Produce NO_2…………………………………………………..2

Supplemental Material, Table S3. Data shown in Figure 2 and Figure 3………………………………..3

References……………………………………………………………………………………..4

Supplemental Material, Table S1. Properties of cooking duration distributions.

	Breakfast			Lunch			Dinner		
	n	GM	GDS	n	GM	GDS	n	GM	GDS
Oven Use	25	15.1	1.8	16	14.4	1.7	138	37.7	1.7
Range Use	163	10.8	1.5	77	14.6	1.9	257	26.9	1.6

The geometric mean (GM) and geometric standard deviation (GSD) of the log-normal distributions from which each cooking event is assigned a duration, in minutes. Results are based on the work of Klug et al. (2011). Due to the limited data, the results were not subdivided into based on the occupancy characteristics of the home.

Supplemental Material, Table S2. Age-based time-location patterns based on NHAPS data (Klepeis et al. 2001).

	Weekday- work at home	Weekday- other	Weekend pattern
0-5 years	Home all the time[a]	If there are senior(s) in home, same as 65+, if not, Away: 8:30 am-5:30 pm	Away: 2pm-4 pm
6-18 years	n/a	Away: 8:30 am-3:00 pm	Away: 2pm-4 pm
19-64 years	Home all the time[a]	Away: 8:30 am- 5:30 pm[a]	Away: 2pm-4 pm
65+ years	n/a	Away: 10-11am	Away: 10-11am

[a]The RASS specifies whether or not a home occupant works at home. If the RASS says an occupant stays home, we assumed one of the 19-64 years olds in the home, as well as any young children (0-5 years) followed the Weekday-stay at home patter. Otherwise, everyone in the home follows the Weekday -other pattern.

Indoor Reactions of NO and Ozone to Produce NO_2

The model did not include homogeneous or heterogeneous chemical reactions, such as the reaction of NO and ozone to produce NO_2. NO emission rates from NGCBs (Singer et al. 2009) are large and available ozone would be the limiting factor in the reaction. To estimate the upper-bound impact of these reactions, we assumed all incoming ozone would react with available NO. Geyh et al (2000) measured ozone concentrations ranging from 5-160 ppb with a mean value of 55 ppb outside of 116 SoCal homes during summer and winter months. Using the average cohort air exchange rate (Summer: 1.2 h^{-1} Winter: 0.44 h^{-1}) and average home size to calculate the effective rate at which ozone would enter the homes, ozone would raise the effective NO_2 emission rate for cooking by 2-72% in summer and by 0-10% in winter depending on location and time of day. This underscores that our estimates for NO_2 concentrations in summer are conservative and that the health impact of NGCB is likely even larger than modeled here.

Supplemental Material, Table S3. Data shown in Figure 2 and Figure 3.

	NO2(ppb) (k=1.05 1/h)[a]										NO2(ppb) (k=0.5 1/h)[a]					CO (ppm)										HCHO (ppb)									
	P5	±%	P25	±%	P50	±%	P75	±%	P95	±%	P5	P25	P50	P75	P95	P5	±%	P25	±%	P50	±%	P75	±%	P95	±%	P5	±%	P25	±%	P50	±%	P75	±%	P95	±%
**Week Avg. (Fig. 2)**																																			
Winter																																			
Concen. outdoors	13	--	19	--	24	--	24	--	28	--	13	19	24	24	28	0.5	--	0.6	--	0.7	--	0.7	--	2.9	--	--	--	--	--	--	--	--	--	--	--
Indoor concen. due to outdoors[b]	3	5	4	2	6	2	8	3	10	3	5	7	10	12	15	0.5	1	0.7	1	0.7	0	0.8	1	2.8	0	--	--	--	--	--	--	--	--	--	--
Total indoor concen.[b]	5	3	8	2	10	3	13	3	21	5	9	12	16	21	34	0.5	2	0.8	1	0.9	3	1.5	5	3.4	4	0	13	0	9	1	7	3	8	13	10
Exposure concen.: No range hood[b]	4	4	7	3	9	4	12	4	22	4	7	11	14	19	33	0.4	4	0.6	2	0.8	3	1.4	7	3.0	9	0	17	0	14	1	8	3	9	13	14
Summer																																			
Concen. outdoors	6	--	8	--	13	--	16	--	16	--	6	8	13	16	16	0.1	--	0.1	--	0.1	--	0.2	--	1.2	--	--	--	--	--	--	--	--	--	--	--
Indoor concen. due to outdoors	2	--	4	--	6	--	8	--	11	--	3	5	8	11	13	0.1	--	0.1	--	0.1	--	0.2	--	1.2	--	--	--	--	--	--	--	--	--	--	--
Total indoor concen.	4	--	6	--	9	--	12	--	17	--	6	9	12	16	24	0.1	--	0.2	--	0.3	--	0.5	--	1.4	--	0	--	0	--	0	--	1	--	6	--
Exposure concen.: No range hood	3	--	6	--	8	--	10	--	16	--	5	8	11	14	23	0.1	--	0.1	--	0.2	--	0.4	--	1.3	--	0	--	0	--	1	--	1	--	6	--
**Peak 1-hr (Fig. 3)**																																			
Winter																																			
Indoor concen. due to outdoors[b]	3	5	5	3	7	3	10	3	14	3	4	8	11	15	20	0.3	4	0.5	1	0.7	3	0.9	2	2.6	4	--	--	--	--	--	--	--	--	--	--
Total indoor concen.[b]	36	4	70	4	110	3	177	6	364	8	46	90	147	229	488	1.0	6	1.9	7	4.2	8	7.9	10	24.2	18	2	11	8	8	19	7	43	8	158	15
Exposure concen.: No range hood[b]	38	6	77	5	127	3	214	4	477	5	49	95	162	279	644	1.0	7	2.0	9	4.5	4	9.0	10	28.1	18	2	12	9	13	21	8	49	9	186	15
Exposure concen.: Range hood on	21	--	39	--	63	--	103	--	218	--	16	49	77	129	288	1.0	--	1.4	--	2.7	--	4.9	--	14.7	--	1	--	4	--	10	--	45	--	95	--
Summer																																			
Indoor concen. due to outdoors	2	--	3	--	5	--	7	--	11	--	2	5	7	9	13	0.0	--	0.1	--	0.1	--	0.2	--	1.4	--	--	--	--	--	--	--	--	--	--	--
Total indoor concen.	27	--	53	--	85	--	139	--	288	--	33	63	102	172	356	0.3	--	1.0	--	2.4	--	4.9	--	18.1	--	1	--	6	--	13	--	30	--	111	--
Exposure concen.: No range hood	30	--	60	--	97	--	164	--	369	--	35	67	113	209	470	0.3	--	1.1	--	2.7	--	5.8	--	21.4	--	1	--	6	--	15	--	36	--	142	--
Exposure concen.: Range hood on	15	--	28	--	46	--	78	--	170	--	20	36	58	101	32	0.2	--	0.5	--	1.3	--	2.6	--	8.6	--	1	--	2	--	6	--	15	--	57	--

[a]Data are shown for NO2 modeled concentrations for both modeled first order loss rates (k=1.05 h-1 and k=0.5 h-1)

[b]Summary statistics for Scenario 1 (winter week, only for k=1.05 h-1 for NO2) are presented as a mean ± range to indicate the variation across the fifteen replicate runs that resulted from reassigning parameter values. Figures 1 and 2 showed the mean values or these runs.

References

Geyh AS, Xue JP, Ozkaynak H, Spengler JD. 2000. The Harvard Southern California chronic ozone exposure study: Assessing ozone exposure of grade-school-age children in two Southern California communities. Environmental Health Perspectives 108:265-270.

Klepeis NE, Nelson WC, Ott WR, Robinson JP, Tsang AM, Switzer P, et al. 2001. The National Human Activity Pattern Survey (NHAPS): A resource for assessing exposure to environmental pollutants. Journal of Exposure Analysis and Environmental Epidemiology 11:231-252.

Klug VL, Lobscheid AB, Singer BC. 2011. Cooking appliance use in California homes – data collected from a web-based survey. LBNL-5028E. Berkeley, CA:Lawrence Berkeley National Laboratory.

Singer BC, Apte MG, Black DR, Hotchi T, Lucas D, Lunden MM, et al. 2010. Natural gas variability in California: Environmental impacts and device performance - experimental evaluation of pollutant emissions from residential appliances. CEC-500-2009-099-APE. Sacramento, CA: California Energy Commision.
